# Radial Distribution Study of Vitreous Barium Borosilicate

**DOI:** 10.6028/jres.067A.006

**Published:** 1963-02-01

**Authors:** G. J. Piermarini, S. Block

## Abstract

An X-ray diffraction study of a barium borosilicate glass consisting of 24 mole percent barium oxide, 40 mole percent boric oxide, and 36 mole percent silicon dioxide has been performed. Resulting atomic radial distribution functions give the following average interatomic distances: Si-O, 1.6 A; Ba-O, 2.8 A; Ba-Ba, 4.7 A; and Ba-Ba, 6.8 A. From the 4.7 A Ba-Ba separation a Ba-O-Ba bond angle of about 115° has been calculated. The observed average barium separations are in partial agreement with that predicted by Levin and Block on the basis of a structural interpretation of immiscibility data. A proposed coordination change by Levin and Block for the barium atoms in the system has been confirmed, but the details of the coordination change mechanism have not.

Combining the results of the radial distribution study and immiscibility data on the barium borosilicate modifier-rich liquid at maximum barium oxide content has indicated that approximately 16.75 mole percent barium oxide is involved in the 4.7 A separation and 8.25 mole percent is associated with the 6.8 A separation.

A mechanism which allows the composition of the modifier-rich liquids in the ternary system to be calculated has been presented. The calculated composition has been found to agree well with the experimental value.

## 1. Introduction

**Figure 1:**
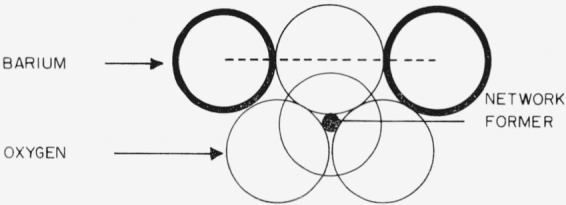
Schematic representation of type A coordination. The network former is in either tetrahedral or triangular configuration. The calculated barium-barium nearest neighbor separation (dash line) is 5.50 Å.

**Figure 2:**
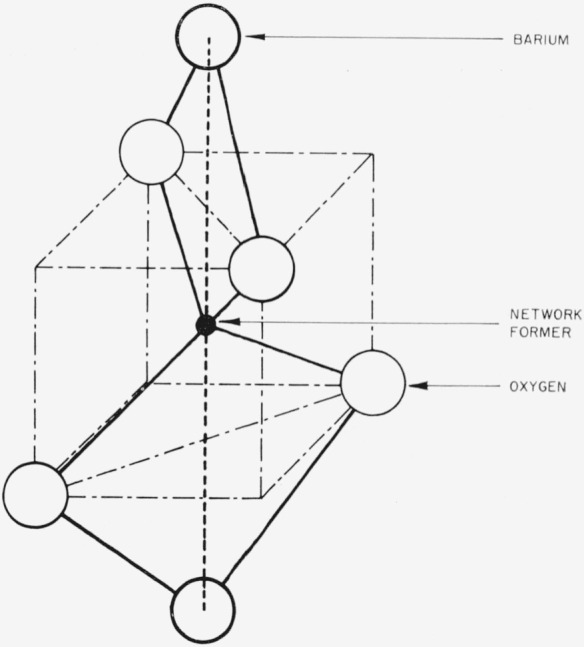
Schematic representation of type B coordination. The network former is in tetrahedral configuration. The network former and barium polyhedra share edges. The calculated barium-barium nearest neighbor separation (dash line) is 6.67 Å.

A comprehensive structural interpretation of the immiscibility of alkali and alkaline-earth metal oxides in borate, silicate, and borosilicate systems has been proposed by Levin and Block [1].^1^ Two models representing short-range structures, originated by Warren and Pincus [2], were adopted to enable the composition of the immiscibility limit to be calculated. The adopted models have been described previously [1,2] and are shown in figures 1 and 2.

For the binary barium borate system at the limit of maximum miscibility of barium oxide in the modifier-rich liquid, Levin and Block proposed that the average separation of barium atoms is 6.67 A resulting from type B coordination. Subsequent radial distribution analysis of the appropriate barium borate glass by Bienenstock, Posner, and Block [3] demonstrated that the proposed model was essentially correct. The average separation of barium atoms was found to be 6.8 A. This conclusion was based on the assumption that the structure of the modifier-rich liquid did not differ appreciably from the structure of the corresponding glassy state obtained by rapid quenching [4].

**Figure 3:**
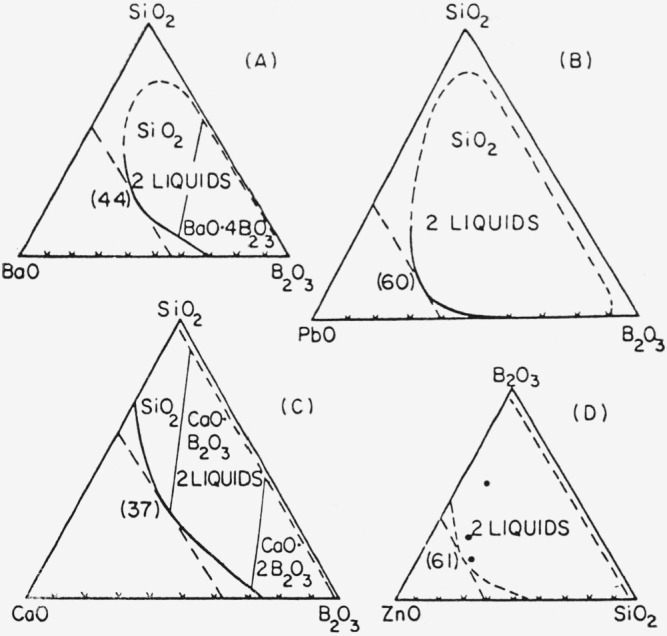
Immiscibility regions (weight percent basis) in systems of type *RO*—*B*_2_*O*_3_-*SiO*_2_ showing form of modifier-rich boundary curves. Numbers in parentheses, adjacent to intersection of solubility curves and tangents, indicate maximum solubility of modifier. To explain the form of these boundary curves, a change in coordination type for the modifier cation has been proposed by Levin and Block. Reproduced from E. M. Levin and S. Block, J. Am. Ceram. Soc. 40, 95 (1957).

For the ternary system, barium borosilicate (part A, fig. 3), it was postulated that the configuration of the barium atoms in the modifier-rich liquids, at the limit of maximum miscibility of modifier oxide, changed progressively upon addition of silicon dioxide component from type B coordination, characteristic of the barium borate system, to type A coordination characteristic of the barium borosilicate system at maximum barium oxide content (see parenthetical number in part (A) of fig. 3 and related legend). Accompanied with the coordination change, a decrease in the average separation of barium atoms from 6.67 A (type B coordination) to 5.50 A (type A coordination) was postulated.

The primary purpose for this research was to compare the average barium interatomic separation in barium borate and barium borosilicate systems at maximum miscibility of barium oxide in the respective modifier-rich liquids in order to determine the mechanism accounting for the observed increase in the immiscible range of barium borosilicate. This paper describes the determination of average interatomic separations by means of a Fourier analysis of X-ray diffraction patterns of the appropriate barium borosilicate glass. It also presents a qualitative interpretation of the observed interatomic distances. With respect to the average barium separation, if the coordination change (B to A) postulated by Levin and Block is a physical reality, then the resulting atomic radial distribution curve should show the presence of a large maximum in the 5.5 A region and the absence of a peak of comparable magnitude in the 6.8 A range.

## 2. Experimental Procedures

The experimental procedures were essentially the same as those employed previously in the vitreous barium borate study [3]. Measurements were made with a Norelco X-ray diffractometer. Intensities were determined with a Norelco counting rate computer adjusted so that the probable intensity error did not exceed 2.4 percent. Monochromatization of the scattered radiation was achieved by means of the balanced filter technique [5]. Intensity correction due to air scatter was avoided by evacuating the volume defined by the scatter shield surrounding the specimen. The intensity data were automatically determined and recorded on a Brown strip-chart recorder at 0.05° intervals in 2θ. Using Cu radiation, measurements were taken in the range 2° to 70° 2θ. With Mo radiation, the intensity measurements were made in the range 20° to 60° 2θ. Periodic checks were made to guard against significant variations in incident intensity. Further detail in the experimental procedure is given in reference 3.

**Figure 4:**
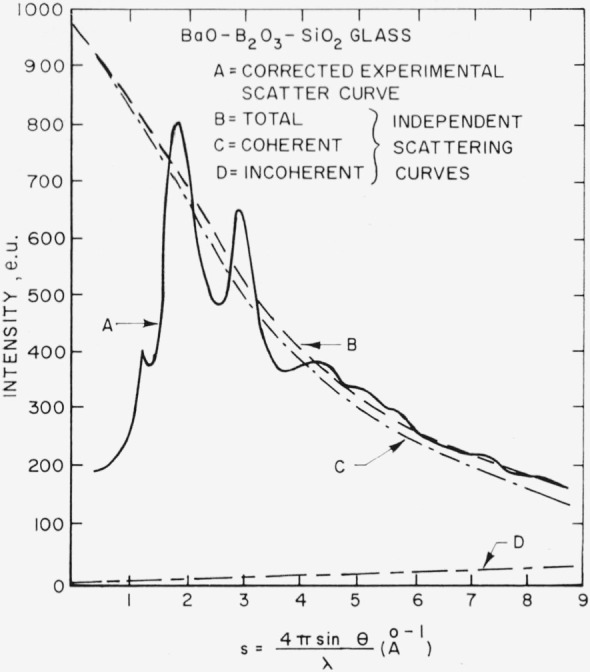
Normalized experimental X-ray intensities (A) and independent scattering curves (B,C,D) for vitreous *BaO*-*B*_2_*O*_3_-*SiO*_2_.

The specimen, containing 24 mole percent barium oxide, 36 mole percent silicon dioxide, and 40 mole percent boric oxide, was obtained from G. W. Cleek and E. H. Hamilton of the National Bureau of Standards staff. The measured density of this specimen, determined by the method described by Glaze, Young, and Finn [6], is 3.35 g cm^−3^. The normalized experimental intensities (A) corrected for polarization are shown in figure 4.

The coherent scattering factors, *f*, for barium, boron, silicon, and oxygen, in addition to the incoherent scattering factors for silicon, boron, and oxygen, were obtained from standard references [7]. The incoherent scattering factor for barium was calculated using [8]

$$I_{\text{inc}}=Z-f^{2}/Z$$

The incoherent scatter was corrected with the Breit-Dirac factor [8]. The total independent scatter curve (B) in figure 4 is the sum of the coherent (C) and incoherent (D) scatter. Unlike the technique employed in the barium borate study [3], the experimental intensities corrected for polarization were put on an absolute scale (electron units per unit of composition) by adjusting the ordinates of the experimental intensity curve so that the experimental curve (A) matched the total independent scattering curve (B) at large s values. This procedure was followed because it appeared less likely to produce high frequency ripples in the distribution curve (assuming that systematic errors in intensity measurements and scattering curves have not been completely eliminated) which would interfere with the determination of peak positions. The method used here tends to push the error residue toward low *s* values thereby producing longer wavelength ripples which interfere less with the determination of peak positions. That high frequency ripples lead to inaccuracies in the distribution curve seems to be indicated in the barium borate study [3]. The normalized intensity curve (A) corrected for polarization is shown in figure 4.

Following essentially the same calculations employed in the previous barium borate study [3], the atomic radial distribution functions were determined. The weighted distribution is usually given by the relation:

$$\sum K_{m}4\pi r^{2}\rho_{m}(r)=\sum K_{m}4\pi r^{2}\rho_{0}+\frac{2r}{\pi }\int_{s_{\min}}^{\infty }si(s)\;\sin\;sr\;ds,$$

where ∑ indicates summation over the molecular composition. *K_m_* is the effective number of electrons in atom *m*, and is defined as *K_m_*=*f_m_*/*f_e_.* 4*πr*^2^*ρ_m_*(*r*)*dr* is the number of atoms, each multiplied by its effective number of electrons, between distances *r* and *r*+*dr* from atom *m*. *ρ*_o_ is the average number of electrons per unit volume, *s* equals (4*π* sin *θ*)/λ, where *θ* is the Bragg angle of diffraction and λ is the wavelength of the radiation. *i*(*s*) is the experimental amplitude function and is equal to $\left(I_{eu}-\sum f_{m}^{2}\right)f_{e}^{2}$, where *I_eu_* is the corrected experimental intensity of unmodified scattering in electron units per unit composition, *f_m_* is the atomic scattering factor for atom *m*, and *f_e_* is the average *f* per electron. That is, *f_e_* equals *∑f_m_*/∑*Z_m_*, where *Z_m_* is the atomic number of atom *m*. The reader is referred to reference 9 for further mathematical detail related to the above general equation.

The determination of the radial distribution function comprises two main steps: First, the numerical evaluation of the *si*(*s*) function from experimental scattering data, and second, the evaluation of the summation

$$\frac{2r}{\pi }\sum_{s_{\min}}^{s_{\max}}si(s)\;\sin\;sr\Delta s.$$

These summations were performed on a high speed digital computer for 0.1 A intervals in *r* from *r*= 0 to *r*=9.9 A over specified ranges in *s*, giving directly the differential radial distribution function,

$$\sum K_{m}4\pi r^{2}[\rho_{m}(r)-\rho_{0}],$$

from which, by adding the average radial atomic density, *∑K_m_*4*πr*^2^*ρ*_0_, the atomic radial distribution as a function of *r* is obtained.

## 3. Results

**Figure 5:**
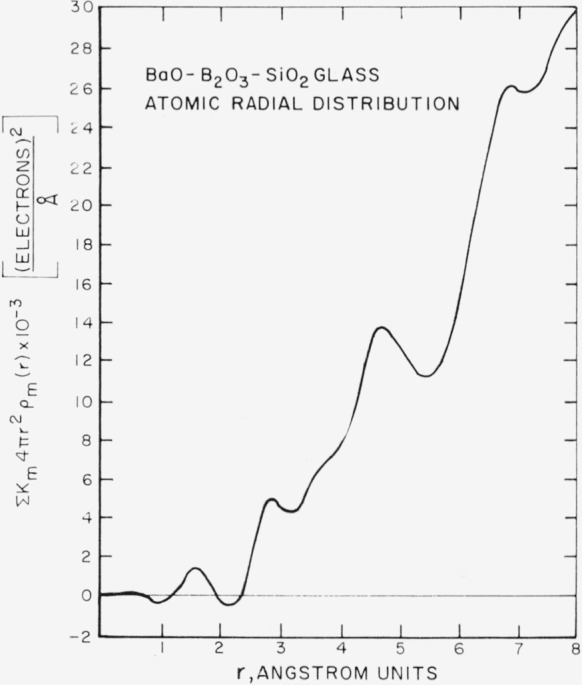
Atomic radial distribution function of *BaO*_2_-*B*_2_*O*_3_-*SiO*_2_ glass.

A fit at high *s* values of the corrected experimental intensity (A), derived from both the molybdenum and copper radiations, to the total independent scatter curve (B) is shown in figure 4. An atomic radial distribution function derived from the experimental intensity data is shown in figure 5. Several techniques were employed in determining the radial distribution function. For example, the series was terminated at various *s* values, the experimental scatter curve (A) was extrapolated to zero at *s*=0, the CuK*_α_* and MoK*_α_* intensity curves were fitted together in several ways, a number of normalization trials were made, and various temperature factors were applied to the *si*(*s*) functions. Application of these techniques produced no significant change in the peak positions in the distribution curve shown in figure 5. However, the peak areas were affected. A temperature factor of $e^{-0.02s^{2}}$ was applied to the experimental amplitude function used in determining the distribution curve shown in figure 5. The intensity data covered the range 0.4 to 8.8 A^−1^ in *s*.

For the purpose of this study the more important features of the distribution function shown in figure 5 are the presence of two unexpected major peaks, one at 4.7 A, the other at 6.8 A, and the absence of an expected major peak in the 5.5 A region. The two observed peaks are attributed primarily to barium-barium interatomic separations, because the barium atom is the only one present in the glass having a large enough scattering factor to produce peaks of this magnitude.

**Figure 6:**
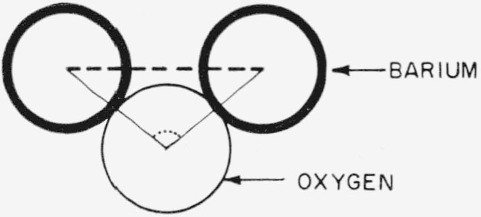
Schematic representation of modified type A coordination depicting the short barium-barium, separation, 4.7 Å, (dash line) and the small barium-oxygen-barium bond angle, 115°, (dot curve). The angle was calculated on the basis of a 2.8 A barium-oxygen separation and the 4.7 A barium-barium separation obtained from the radial distribution curve.

The 4.7 A barium-barium separation is associated with a modified type A coordination wherein the barium-oxygen-barium bond angle is approximately 115, not 180° as postulated by Levin and Block. A schematic representation of modified type A coordination is shown in figure 6. If it is assumed that the barium atoms form, in general, a cubic array with an average edge of 4.7 A, then the face diagonal of this cube has a length of 6.65 A. Although the radial distribution function shows high atomic density in the region of 6.65 A, the maximum is at 6.8 A. This peak is primarily due to barium atoms in type B coordination, and the second nearest neighbor barium-barium separation arising from modified type A coordination. A similar barium separation (6.8 A) was found for type B coordination in the barium borate study by Bienenstock, Posner, and Block [3].

Further analysis of the distribution function indicates that the 1.6 A peak represents primarily the average silicon-oxygen nearest neighbor distance. The calculated distance is 1.62 A. By a similar radial-distribution study, Warren et al., obtained 1.62 A for the silicon-oxygen nearest neighbor distance in silica glass [9]. The 2.8 A peak represents the barium-oxygen nearest neighbor distance. The calculated separation is 2.8 A.

Coordination numbers calculated from the observed areas of maxima are rather doubtful due to systematic errors and the overlap of interatomic separations inherent in the technique. In addition, for complex glasses such as barium borosilicate, the calculation of coordination numbers is complicated.

Consider the 1.6 A peak. There are three interatomic separations contributing to the area of this peak. One is the previously mentioned 1.6 A silicon-oxygen separation, another is a 1.4 A boron-oxygen separation resulting from triangularly coordinated boron, and also present is a 1.5 A boron-oxygen separation arising from tetrahedrally coordinated boron [10]. The contributions of the two boron-oxygen separations to the area of this peak are indeterminate. However, compared with the contribution of the 1.6 A silicon-oxygen separation, the contributions due to the boron-oxygen separations are minor because of the low atomic number of boron. With due consideration for these facts, an estimated area of 400 (electrons)^2^ is attributed to the 1.6 A silicon-oxygen separation. From this area a coordination number of 4.3 is calculated for the number of oxygen nearest neighbors about a silicon atom (see reference 3 for coordination number calculations). This value is consistent with the predicted value, 4.

The area of the 2.8 A peak has contributions from several interatomic separations as in the previous case. The primary contribution to this peak is from the barium-oxygen nearest neighbor separation. The area of the 2.8 A peak is estimated to be 2,500 (electrons)^2^. If one were to assume 8-fold coordination for the oxygen nearest neighbors about a barium atom, then the calculated area is approximately 1,500 (electrons)^2^.

The more important aspects of this research, the coordination change and estimate of percent of barium atoms in either coordination grouping, can be treated further. Continuing with the previous assumption that the barium atoms form a cubic array, an average barium separation of 5.58 A is calculated using the Warren and Pincus equation which states that the extent of immiscibility is inversely proportional to the cube of the modifiercation separation. The radial distribution function does not allow a determination of the relative amounts of barium atoms in type B and modified type A coordination but, as the contributing distances and average distance are known, the following calculation can be made to provide an estimate of the fraction of barium atoms in each coordination type.

Letting *α* equal the fraction of barium atoms involved in type B coordination and (1*—α*) the fraction involved in modified type A coordination, then

$$\begin{array}{c} \alpha {\left(6.8\;A\right)}^{3}+(1-\alpha ){\left(4.7\;A\right)}^{3}={\left(5.58\;A\right)}^{3}\\ \alpha =0.33. \end{array}$$

Since the total amount of barium oxide in the modifier-rich liquid is 25 mole percent, then approximately 8.25 mole percent is associated with type B coordination and about 16.75 mole percent is involved in modified type A coordination.

The ratio of mole percents of barium oxide (16.4%) to boric oxide (83.6%) in the binary barium borate system at the limit of miscibility of barium oxide in the modifier-rich liquid is approximately 0.20. For this system the barium atoms have the type B configuration [3]. It is interesting to note that the ratio of mole percents of barium oxide (8.25%) to boric oxide (40%) for the corresponding modifier- rich liquid in the ternary barium borosilicate system is 0.21, when only the calculated amount of barium oxide involved in type B coordination is considered. The similarity of ratios suggests that the amount of barium atoms in type B coordination remains constant as silica and barium oxide components are added, while the added barium atoms assume the modified type A coordination.

Using the oxygen-volume method [1] the composition of the maximum extent of miscibility of modifier oxide can be estimated. For barium borate in type B coordination, the relative mole percentages are: 16.1 BaO·83.9 B_2_0_3_ [1]. For barium silicate in modified type A coordination, the relative mole percentages are: 32.2 BaO·67.8 SiO_2_. The combined compositions can be written as: 32.2 BaO (modified type A)·16.1 BaO (type B)·83.9 B_2_O_3_·67.8 SiO_2_ or on a 100 percent basis as: 16.1 BaO (modified type A) · 8.0 BaO (type B) · 42.0 B_2_O_3_·33.9 SiO_2_. This composition compares well with the experimental composition, 16.75 BaO (modified type A) · 8.25 BaO (type B)·39 B_2_O_3_·36 SiO_2_, where the relative amounts of each coordination type were determined by the calculation of *α*, the fraction of barium atoms involved in type B coordination.

## 4. Conclusions

The radial distribution functions reveal barium-barium separations at approximately 4.7 and 6.8 A. The unexpected 4.7 A distance is due to barium atoms in a modified type A coordination with a calculated barium-oxygen-barium bond angle of about 115°. The 6.8 A distance is due to barium atoms in type B coordination and the barium-barium second nearest neighbor separation due to modified type A coordination. The new factor as compared to the average barium separation in the barium borate glass is the 4.7 A separation. As postulated by Levin and Block, the increase of the immiscibility range in the ternary barium borosilicate system does occur by a coordination-change mechanism. However, the barium atoms are not involved solely in type A coordination. The factor which caused Levin and Block to assume 100 percent change to type A coordination is the good agreement observed between the average barium-barium separation (5.58 A) determined by using the Warren and Pincus immiscibility equation and the geometrical separation (5.50 A) due to the type A configuration with a 180° barium-oxygen-barium bond angle.

At the limit of miscibility in the binary barium borate system, the modifier-rich liquid contains 16.4 mole percent barium oxide with the barium atoms involved primarily in type B coordination. The corresponding barium borosilicate modifier-rich liquid contains 25 mole percent barium oxide [1]. Combining the results of the radial distribution study and the immiscibility equation indicates that approximately 16.75 mole percent barium oxide is involved in the modified type A configuration and 8.25 mole percent barium oxide is associated with type B coordination. In the barium borate modifier-rich liquid as silica and barium oxide components are added, it may be considered that the amount of barium atoms in type B coordination remains constant, while the additional barium atoms assume a modified type A coordination. Using the modified type A coordination model and the oxygen-volume method of calculating the extent of miscibility of modifier oxide [1] results in a final limiting composition of 16.1 BaO (modified type A)·8.0 BaO (type B)·42.0 B_2_0_3_·33.9 SiO_2_ in mole percent.

Each coordination type, modified A or B, is not necessarily associated with a particular network former. One should consider the structure with type B coordination as having modifier cations separated primarily by the network former as described earlier in figure 2 and related legend. Thus, the modifier cations are bonded to different oxygens. The structure with modified type A coordination should be considered as having modifier cations bonded primarily to the same oxygen atom.

This study indicates the importance of determining whether the phenomenon of coexistence of type B and modified type A coordinations is limited to the ternary system. Research on relevant binary systems is presently being undertaken.

## Footnotes

1 Figures in brackets indicate the literature references at the end of this paper.
